# 578. The Power of the Shower: The Importance of Having a Sustainable Emergency Department Decontamination Process

**DOI:** 10.1093/jbcr/irag033.341

**Published:** 2026-04-06

**Authors:** Steve Elmgren, Carey K Lamphier, Lori M Wood, Brooks L Moore, Pamela Vanderberg, Kristen Glasgow-Petela, Yuk Ming Liu, Laura S Johnson, Lauren B Nosanov

**Affiliations:** Grady Memorial Hospital, Atlanta, GA; Grady Memorial Hospital, Atlanta, GA; Grady Health System, Atlanta, GA; Grady Memorial Hospital, Atlanta, GA; Grady Memorial Hospital, Atlanta, GA; Morehouse School of Medicine, Decatur, GA; Emory University School of Medicine, Atlanta, GA; Grady Memorial Hospital / Emory School of Medicine / Morehouse School of Medicine, Atlanta, GA; Emory University School of Medicine, Atlanta, GA

## Abstract

**Introduction:**

Decontamination (decon) plays a key role in optimal emergency management of the burn patient and patient outcomes by “stopping the burn” and protecting healthcare providers from secondary exposure. In summer 2023, our large, urban, verified burn and Level I trauma center received pre-notification of a patient with chemical burn injuries. Initially reported that decon was done at the scene, it became evident upon arrival that it was incomplete. While transferring the patient to the designated decon area, several zones in the trauma center were exposed to the chemical, resulting in potential staff exposure and disruption of critical hospital and community resources. This incident revealed opportunity and stressed the importance of a structured and consistent decon process.

**Methods:**

A multidisciplinary After Action Review met to analyze the event, assess existing processes, and identify system-level challenges. While the review confirmed key resources (decon facilities, notification platforms, and subject matter experts) were already in place, it showed they were not fully leveraged. This prompted a full evaluation of current policies and procedures, engaging a broad group of stakeholders to enhance decon readiness and response.

**Results:**

A multidisciplinary work group from the Emergency Department, Emergency Management, Burn Surgery, and Toxicology was established. Extensive education and training initiatives were implemented including developing "superusers" and a dedicated decon team to ensure consistent and skilled response capabilities. Existing decon policies were revised to clarify indications, responsible personnel, and standard procedures. Full-scale exercises were conducted to test and refine new processes. Tracking and reporting metrics were introduced for the Burn Process Improvement team to monitor all burn-related decon events, enabling ongoing evaluation and quality improvement.

**Conclusions:**

Much of the revised decon process is functioning well, including effective pre-hospital notifications and activation of internal paging systems, allowing for timely mobilization of key response teams. The dedicated team was another success, largely due to strong frontline staff engagement. Staffing remains a challenge due to normal attrition, impacting the ability to have enough trained staff across all shifts, underscoring the need for ongoing training. There is still a need for more robust documentation which includes working closely with the electronic medical record team to optimize workflows and nursing staff to reinforce thorough documentation; formalization of documentation policies will help standardize practices and ensure consistency across providers. We continue to review and revise procedures to ensure appropriate universal protocols.

**Applicability of Research to Practice:**

Timely mobilization, specialized team members, and consistent review all support reduced risk, improved care, and efficient practice.

**Funding for the study:**

N/A.

